# Role of 18F-Fluorocholine Positron Emission Tomography (PET)/Computed Tomography (CT) in Diagnosis of Elusive Parathyroid Adenoma

**DOI:** 10.7759/cureus.48892

**Published:** 2023-11-16

**Authors:** Janan R Badier, Pokhraj P Suthar, Jagadeesh S Singh, Miral D Jhaveri

**Affiliations:** 1 Diagnostic Radiology & Nuclear Medicine, Rush University Medical Center, Chicago, USA; 2 Diagnostic Radiology and Nuclear Medicine, Rush University Medical Center, Chicago, USA

**Keywords:** 18f-fluorocholine pet, spect/ct, mri, 4d-ct, parathyroid

## Abstract

Accurate localization of parathyroid adenomas is paramount in hypercalcemia and elevated parathyroid hormone (PTH) levels. This narrative of a 56-year-old female diagnosed with primary hyperparathyroidism underscores the intricacies faced when conventional imaging falls short. Despite a series of diagnostic and surgical endeavors, including an initial nuclear sestamibi scan and diverse imaging examinations like ultrasound, 4D CT, and MRI, it was the 18F-Fluorocholine positron emission tomography (PET)/computed tomography (CT) scan that illuminated the presence of the elusive adenoma in the left para esophageal superior mediastinum. The surgical outcome reinforced the diagnosis, marking the resolution of the adenoma. This case accentuates the necessity of a multifaceted diagnostic methodology, especially in convoluted primary hyperparathyroidism presentations. It highlights the yet-to-be widely adopted 18F-Fluorocholine PET/CT scan, emphasizing its prospective significance awaiting Food and Drug Administration (FDA) endorsement.

## Introduction

In the context of hypercalcemia with concomitantly normal or elevated parathyroid hormone (PTH) levels, differential diagnoses encompass primary hyperparathyroidism, familial hypocalciuric hypercalcemia, and the effects of lithium therapy [1]. Primary hyperparathyroidism, commonly attributed to parathyroid hyperplasia or adenoma, mandates parathyroidectomy as the definitive therapeutic intervention [2]. Various imaging techniques, such as ultrasonography, technetium sestamibi integrated with single-photon emission computed tomography (SPECT)/CT, C-methionine positron emission tomography (PET), and 4D CT, have proven efficacious for adenoma identification. Emerging in the imaging horizon is the choline PET scan, employing either 18F-choline or 11C-choline, showcasing potential in pinpointing adenomas undetected in preceding imaging or surgical endeavors. Nonetheless, its current status remains outside Food and Drug Administration (FDA) approval, limiting its accessibility across the United States [3].

## Case presentation

**Figure 1:**
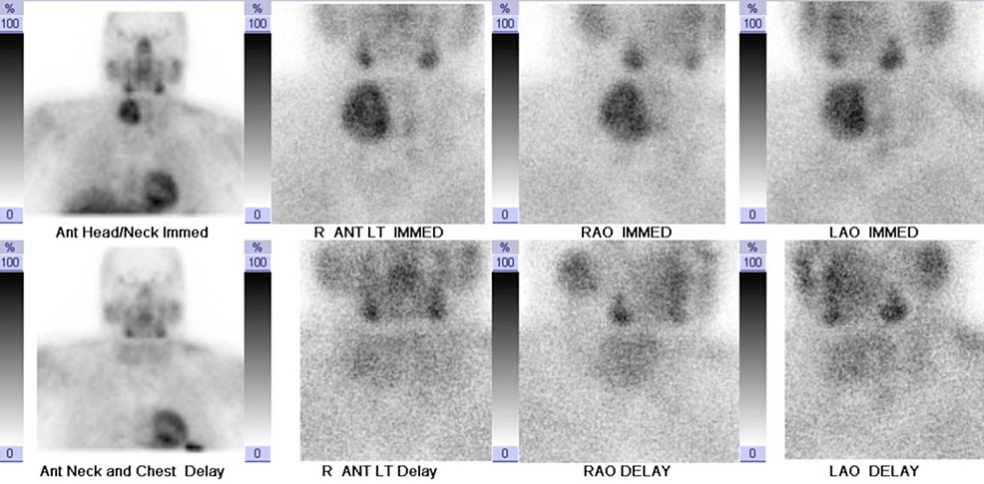
(Upper row) Planar initial 15-minute image and (lower row) 2.5-hour delayed planar image from first nuclear medicine parathyroid scintigraphy after injection of 24.5 millicuries of technetium-labeled sestamibi. The initial image demonstrates asymmetrical increased tracer uptake in the right thyroid region. The delayed image does not show abnormal tracer retention to suggest parathyroid adenoma.

**Figure 2:**
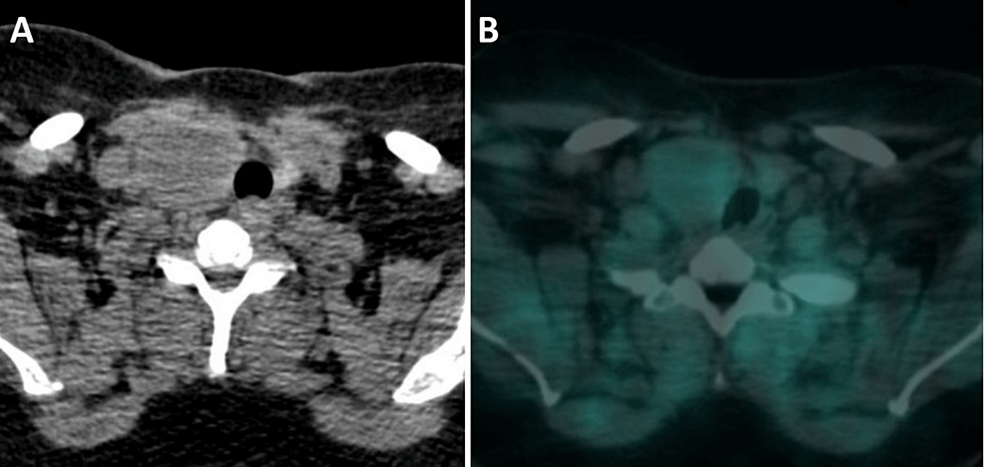
The 3-hour delayed (A) unfused axial CT and (B) fused axial SPECT/CT images from the first parathyroid scintigraphy reveal demonstrates asymmetrical enlarged right thyroid lobe. No abnormal tracer retention suggestive of a parathyroid adenoma.

**Figure 3:**
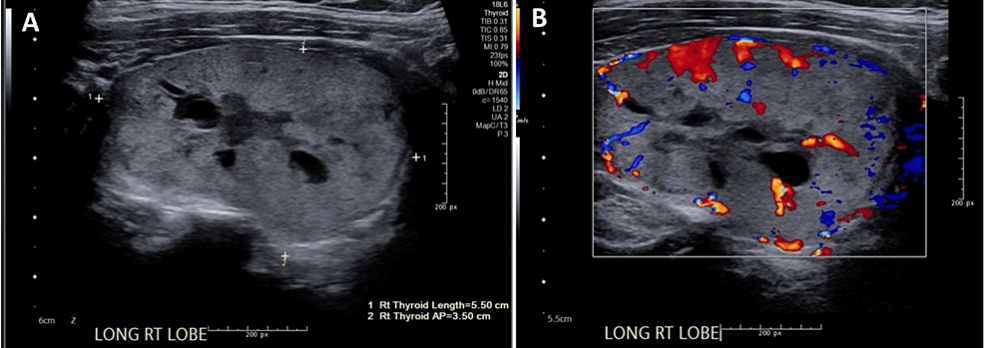
(A) Ultrasound and (B) color duplex of the right thyroid shows 5.1 x 2.9 x 3.5 cm biopsy proven TIRADS category 2 right thyroid nodule.

A 56-year-old female with a history of hypothyroidism and type 2 Diabetes mellitus exhibited hypercalcemia and hyperparathyroidism (intact PTH level 181.9 pg/mL; reference range 8.0 - 85.0 pg/mL) on routine blood investigations. She was evaluated at a tertiary academic hospital's Ear Nose Throat (ENT) department, where she reported a 10-pound weight increase over three months, accompanied by fatigue and increased frequency of loose stools. No familial thyroid or parathyroid disorders were documented. The first nuclear medicine sestamibi scan with SPECT/CT showed an asymmetrical enlargement of the right thyroid lobe without focal tracer retention suggestive of a parathyroid adenoma (Figures 1, 2). Subsequently, the patient underwent a right thyroid fine-needle aspiration (FNA) and a thyroid ultrasound (Figure 3), which revealed a benign right thyroid nodule. The patient subsequently underwent a right hemithyroidectomy with surgical exploration of all four parathyroid glands. Unfortunately, only three parathyroid glands were identified despite a comprehensive surgical examination, leading to a parathyroidectomy of the three identified glands. Despite these interventions, her hyperparathyroidism persisted, evidenced by consistently elevated PTH levels and fluctuating postoperative ionized calcium measurements. Histopathology revealed hyperplastic adenomatous changes in the right thyroid nodule, benign lymph nodes, and three normocellular parathyroid glands. Consequently, several potential ectopic sites were proposed.

**Figure 4:**
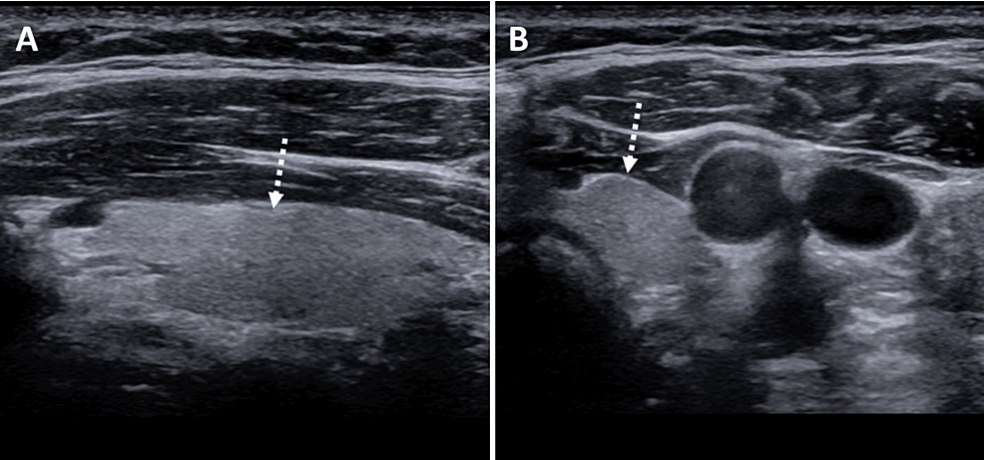
Ultrasound of the soft tissue of the neck (A) longitudinal and (B) transverse views of the left thyroid lobe shows an unremarkable left thyroid lobe (dashed white arrows) with no ultrasound evidence of a parathyroid nodule.

**Figure 5:**
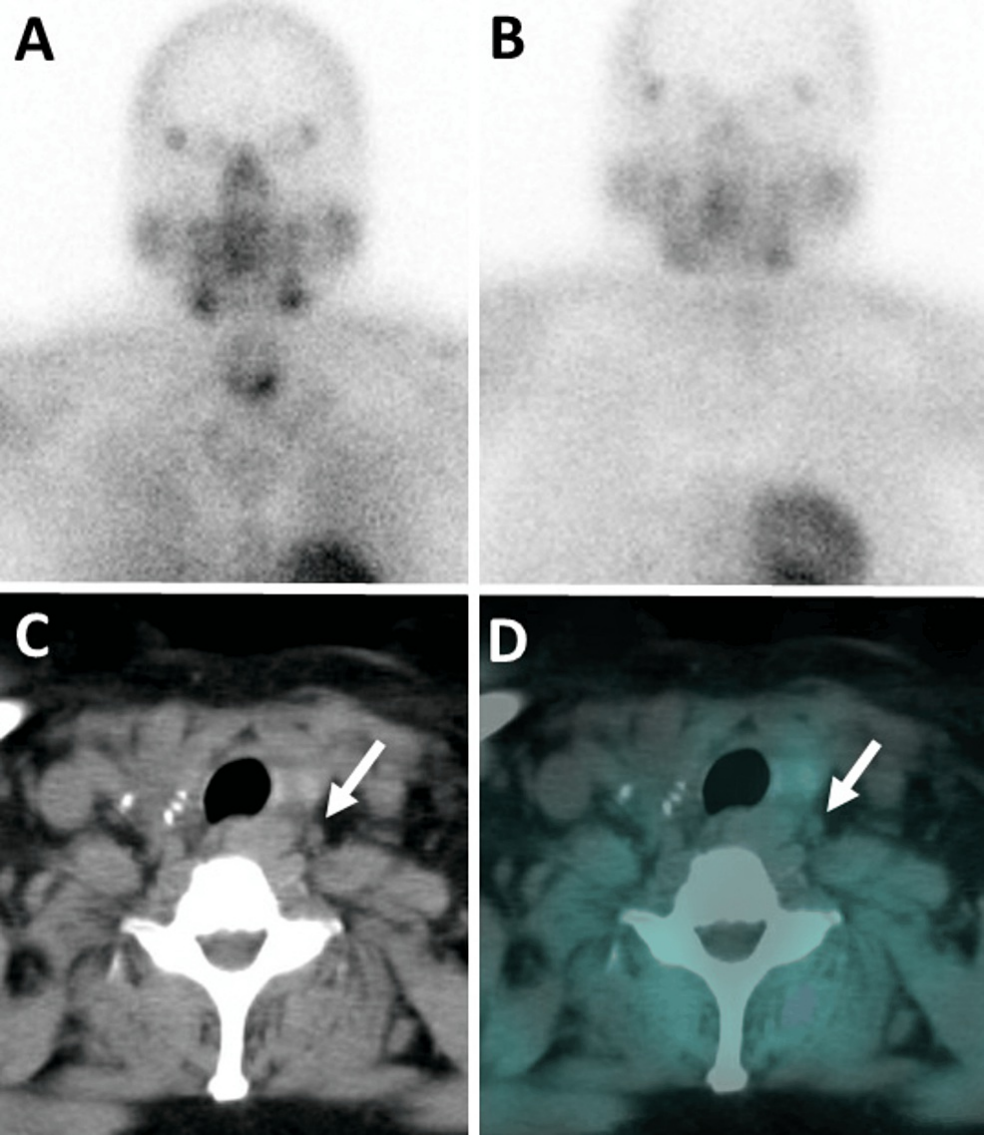
Nuclear medicine parathyroid scintigraphy. (A) Planar initial 15-minute image and (B) 2.5-hour delayed planar image after injection of 25.4 millicuries of technetium-labeled sestamibi. The initial image demonstrates mild tracer uptake in the right thyroid bed, consistent with residual thyroid tissue. There is asymmetrical increased tracer uptake in the left thyroid lobe. The delayed image does not show abnormal tracer retention, suggestive of a parathyroid adenoma. The 2.5-hour delayed (C) unfused axial CT and (D) fused axial SPECT/CT images from the parathyroid scintigraphy reveal an absence of tracer uptake in the 0.4 x 0.3 cm left paraoesophageal superior mediastinal soft tissue nodule (white arrow), suggesting it is unlikely to be a parathyroid adenoma.

Ultrasound of the soft tissue neck showed an unremarkable left thyroid lobe, devoid of parathyroid nodules (Figure 4). A re-examination using the nuclear sestamibi scan was performed. The initial image demonstrates mild tracer uptake in the right thyroid bed, consistent with residual thyroid tissue. There was asymmetrical increased tracer uptake in the left thyroid lobe. The delayed image did not show abnormal tracer retention, suggestive of a parathyroid adenoma. The 2.5-hour delayed SPECT/CT images from the parathyroid scintigraphy reveal an absence of tracer uptake in the left para esophageal superior mediastinal soft tissue nodule, suggesting it is unlikely to be a parathyroid adenoma (Figure 5).

**Figure 6:**
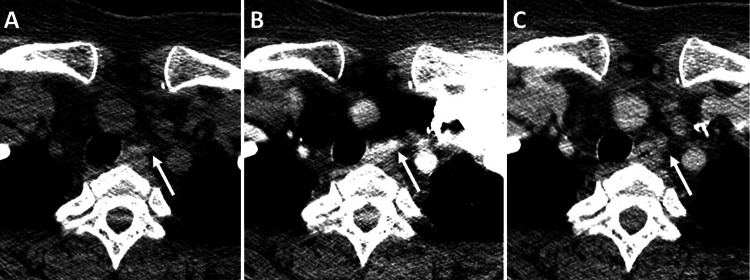
The 4D CT neck. (A) Non-contrast, (B) arterial phase, and (C) venous phase after the administration of 100 ml of intravenous contrast Iopamidol 76% (Isovue 370, Bracco) into the left upper extremity. Streak artifacts from the venous contrast limit accurate evaluation. However, there is a 6 mm soft tissue nodule in the superior mediastinum on the left ventral to the esophagus (white arrows). This nodule demonstrates suspected enhancement in the arterial phase with washout in the venous phase, raising concerns for a suspicious parathyroid adenoma.

**Figure 7:**
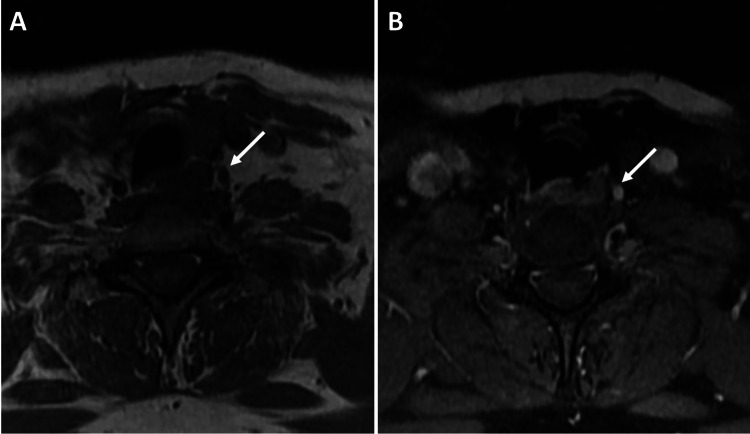
MRI of the soft tissue neck. (A) Axial T1 non-contrast and (B) axial T1 fat-saturated post-contrast images following the intravenous injection of 20 mL of gadoteridol (ProHance; Bracco). These images reveal a subcentimeter T1 isointense enhancing nodule in the left paraesophageal region of the superior mediastinum (white arrows), which correlates with the suspicious nodule observed in the 4D CT.

**Figure 8:**
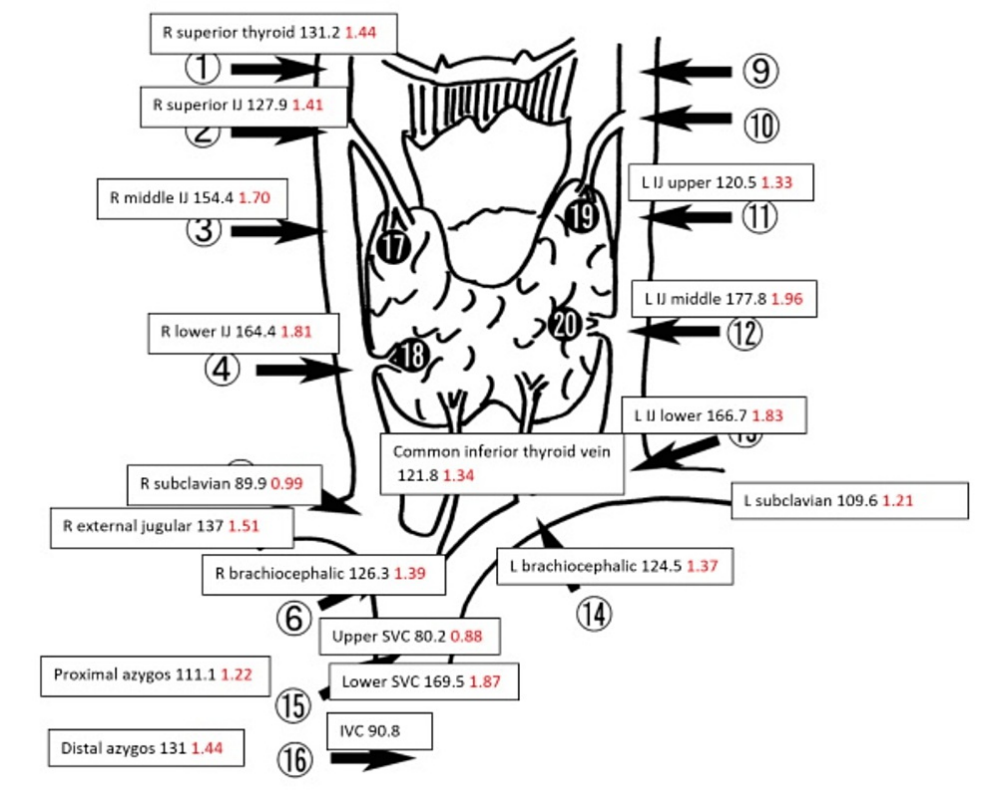
Interventional radiology (IR) parathyroid venous mapping results demonstrate inconclusive localization due to the confounding factor of cinacalcet. The iPTH value in pg/ml is denoted in black numbers at the respective venous sampling sites (for example, the right superior thyroid vein iPTH value is 131.2 pg/ml). The iPTH ratio (ratio of iPTH value at the respective venous sampling site relative to the inferior vena cava iPTH level) denoted in red numbers in respective venous sampling site (for example, the right superior thyroid vein iPTH ratio is 1.44).

**Figure 9:**
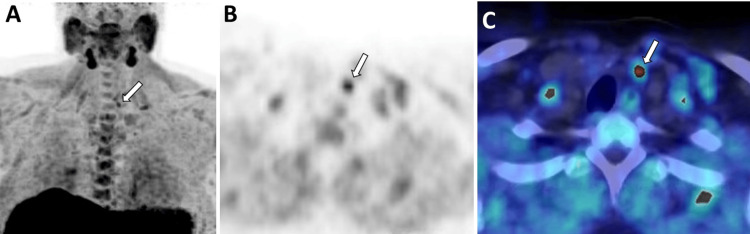
18F-Fluorocholine PET/CT. (A) Maximum intensity projection (MIP), (B) unfused axial PET, and (C) fused axial PET/CT images after an injection of 4.01 mCi of 18F-Fluorocholine display a subcentimeter focus of increased tracer uptake (SUVmax 4.5) in the left paraesophageal superior mediastinum (white arrow), consistent with a parathyroid adenoma.

A subsequent 4D CT scan was significantly limited due to streak artifacts from the venous contrast injection; however, a suspicious nodule was observed in the para esophageal superior mediastinum (Figure 6). A neck MRI, albeit affected by pulsation artifacts, confirmed a subcentimeter T1 isointense enhancing nodule in the left para esophageal superior mediastinum, which can be either a lymph node or parathyroid nodule (Figure 7). Parathyroid venous sampling was conducted to pinpoint the adenoma's location. Notably, the maximum ratio was detected at the left thyroid bed, but this lacked statistical significance. Significantly, the patient's continued use of cinacalcet might have influenced these findings (Figure 8). Due to the inconclusiveness of previous imaging, the patient sought an F18 choline PET scan in a different state at an institution with FDA approval for 18F-Fluorocholine PET/CT. The 18F-Fluorocholine PET/CT revealed a subcentimeter focus with heightened tracer uptake (SUVmax 4.5) in the left para esophageal superior mediastinum, consistent with a parathyroid adenoma (Figure 9). 

Another surgical intervention identified a left parathyroid adenoma encapsulated within fibrofatty tissue, positioned beneath the left thyroid lobe and anterior to the esophagus in the superior mediastinum. Around 70% of the adenoma was removed, leaving 30% intact. Post-surgery, a notable decrease in iPTH levels to 4.7 was observed, conclusively indicating the resolution of primary hyperparathyroidism. The patient's postoperative period was smooth, and she reported no symptoms of iatrogenic hypocalcemia, such as perioral numbness or muscle spasms.

## Discussion

The parathyroid glands, diminutive endocrine structures adjacent to the thyroid, typically weigh around 0.5 g [4]. In 85% of individuals, there are four parathyroid glands: superior and inferiorly positioned [5]. The superior glands originate embryologically from the fourth branchial pouch, whereas the inferior ones descend embryologically with the thymus [6]. This embryological descent path determines the potential sites for ectopic inferior glands, most commonly situated in the anterior mediastinum. Conversely, superior glands are frequently ectopically located in the tracheoesophageal groove and gastroesophageal region. Anatomical studies have shown a 2-43% prevalence of ectopic parathyroid glands, and this incidence rises to 16% in those diagnosed with primary hyperparathyroidism [7].

Parathyroid adenomas encompass a spectrum from hyperplasia to carcinoma, primarily affecting individuals aged 50-70 [8]. Of hyperparathyroidism cases, 80-85% arise from single adenomas, 10-12% result from parathyroid hyperplasia, 4-5% from dual adenomas, and a mere <1% from parathyroid carcinoma. The condition disproportionately affects women at rates thrice that of men [9]. Tumors weighing under 0.1 g are termed microadenomas, while those exceeding 2.0 g are labeled giant adenomas [6]. These neoplasms often secrete excessive parathyroid hormone, leading to hyperparathyroidism.

The unregulated overproduction of parathyroid hormone typifies primary hyperparathyroidism (HPT). It can be sporadic or familial, with familial causes including multiple endocrine neoplasia (MEN) types 1 and 2, non-MEN familial hyperparathyroidism, and hyperparathyroidism-jaw tumor syndrome. However, in most instances, the adenoma's etiology remains elusive. Cyclin D1/PRAD1 gene mutations emerge as the predominant cause of sporadic adenomas. External factors, such as radiation therapy, prolonged lithium use, and chronic calcium deficiency, also contribute to the formation of these adenomas [10].

Hypercalcemia-related symptoms, such as neuropsychiatric manifestations, bone pain, fatigue, constipation, polyuria, and nephrolithiasis, are common presentations for those harboring a parathyroid adenoma. Severe calcium elevation can precipitate cardiac arrhythmias, coma, or even fatal outcomes. Distinct from thyroid neoplasms, palpation seldom identifies parathyroid adenomas during physical examinations. Incidental hypercalcemia detection on routine bloodwork leads to a subsequent PTH level evaluation [10].

While most adenoma patients exhibit mild hypercalcemia-about 1.0 mg/dL above the standard range, they also display inappropriately elevated or within-range PTH levels. Following biochemical confirmation, imaging investigations become imperative. Intrinsically, normal parathyroid glands remain elusive on ultrasound; thus, any visibility on this modality should heighten suspicion. However, ultrasound's sensitivity is 60-80%, making a negative result inconclusive. The reigning diagnostic gold standard is the fusion of SPECT with technetium-99m (99mTc) sestamibi scintigraphy, boasting a 91-98% sensitivity for single adenomas [9]. Alternatively, employing a dual radioisotope strategy with 99mTc pertechnetate and thallium-201 facilitates a subtraction imaging approach, enhancing visualization. The existence of cysts within parathyroid proliferative lesions, along with the small size (<2 cm) and multiple number of parathyroid lesions, can lead to false negatives in parathyroid methoxy isobutyl isonitrile (MIBI) scans [11, 12]. 4D-CT predicted the correct side of single-gland adenomas in 93% of cases [9]. Multiparametric MR perfusion can accurately differentiate parathyroid adenomas from adjacent thyroid tissue or lymph nodes, achieving a diagnostic accuracy rate of 96% [13].

Parathyroidectomy stands as the treatment of choice for primary hyperparathyroidism. However, familial hypocalciuric hypercalcemia is an unequivocal contraindication and contralateral recurrent laryngeal nerve injury is a relative contraindication. Factors contributing to the failure to identify parathyroid disease include challenges in locating and excising ectopically positioned adenomas, particularly those in the mediastinum, the presence and persistence of supernumerary glands, errors in frozen-section examinations, and incomplete excision of invasive parathyroid carcinoma [14]. In our case, the patient's clinical presentation was complex due to comorbid conditions, and the lack of clear localization could have potentially resulted in a less precise initial surgical procedure. 

Potential postoperative complications range from recurrent laryngeal nerve injury and ensuing hypoparathyroidism leading to hypocalcemia to grave hemorrhage. A retrospective analysis revealed a life-threatening hematoma in six out of 1,050 thyroidectomy and parathyroidectomy patients [15]. Voice changes, stridor, and potential airway obstruction due to bilateral nerve impairment are recurrent laryngeal nerve injury symptoms. However, recovery often ensues by the six-month postoperative mark [16]. Most postoperative hypocalcemia cases are transient; a paltry 0.5-3.8% persist. Short-term hypocalcemia can be managed with calcium and synthetic vitamin D, while prolonged cases may necessitate supplemental PTH therapy [17].

Persistent hyperparathyroidism post-parathyroidectomy is not an uncommon phenomenon. Defined as unable to achieve normocalcemia within half a year post-procedure, this condition afflicts 2-5% of sporadic primary hyperparathyroidism patients. This complication is more frequent in the backdrop of an ectopic adenoma or multiple adenoma. The typical recourse in these scenarios is re-operation [17].

In the past, bilateral neck exploration dominated the therapeutic landscape. However, the ascent of minimally invasive parathyroidectomy (MIP) has altered this paradigm. For MIP to triumph, preoperative pinpointing of the adenoma is essential [4]. Traditional imaging tools, such as nuclear imaging 99mTc sestamibi scintigraphy or ultrasound, facilitate this. Nevertheless, ultrasound efficacy diminishes when confronted with ectopic glands. In such intricate cases, the amalgamation of 11 C-choline or 18 F-choline PET/CT or parathyroid venous sampling can be invaluable. Radiolabeled choline PET, utilizing either 11 C-choline or 18 F-choline, is a burgeoning modality explored for parathyroid adenoma detection. A study encompassing 35 primary hyperparathyroidism patients bestowed 18 F-choline PET with a sensitivity and specificity of 96% and 100%, respectively. Conversely, technetium-99 SPECT managed a sensitivity of 78% [18]. The paramount diagnostic prowess of 18-F choline is tempered by its prohibitive cost, scarce availability, and lack of FDA clearance for primary hyperparathyroidism diagnosis [19].

## Conclusions

The diagnostic trajectory for primary hyperparathyroidism is frequently intricate, particularly when traditional imaging modalities prove inadequate in accurately localizing the adenoma. This case elucidates the salient potential of adopting an integrated diagnostic paradigm, leveraging diverse imaging modalities to optimize adenoma localization. Though pending FDA endorsement, the 18F-Fluorocholine PET/CT exhibited superior efficacy in this context. Its pivotal contribution in the definitive identification of the adenoma, subsequent surgical guidance, and the resultant amelioration of the patient's primary hyperparathyroidism underscores its prospective significance in the clinical domain.
